# Assessment of Three Mitochondrial Genes (16S, Cytb, CO1) for Identifying Species in the Praomyini Tribe (Rodentia: Muridae)

**DOI:** 10.1371/journal.pone.0036586

**Published:** 2012-05-04

**Authors:** Violaine Nicolas, Brigitte Schaeffer, Alain Didier Missoup, Jan Kennis, Marc Colyn, Christiane Denys, Caroline Tatard, Corinne Cruaud, Catherine Laredo

**Affiliations:** 1 Muséum National d'Histoire Naturelle, Département de Systématique et Evolution UMR CNRS 7205, Paris, France; 2 INRA, UR341 Mathématiques et Informatique Appliquées, Jouy-en-Josas, France; 3 Department of Animal Biology Organisms, Faculty of Science, University of Douala, Douala, Cameroon; 4 Evolutionary Ecology Group, University of Antwerp, Antwerpen, Belgium; 5 Université de Rennes 1, UMR CNRS 6553 Ecobio, Paimpont, France; 6 Centre de Biologie et de Gestion des Populations, UMR IRD 022, Montferrier-sur-Lez, France; 7 Genoscope, Centre National de Sequençage, Evry, France; 8 Université Denis Diderot, LPMA UMR 7599, Paris, France; Biodiversity Insitute of Ontario – University of Guelph, Canada

## Abstract

The Praomyini tribe is one of the most diverse and abundant groups of Old World rodents. Several species are known to be involved in crop damage and in the epidemiology of several human and cattle diseases. Due to the existence of sibling species their identification is often problematic. Thus an easy, fast and accurate species identification tool is needed for non-systematicians to correctly identify Praomyini species. In this study we compare the usefulness of three genes (16S, Cytb, CO1) for identifying species of this tribe. A total of 426 specimens representing 40 species (sampled across their geographical range) were sequenced for the three genes. Nearly all of the species included in our study are monophyletic in the neighbour joining trees. The degree of intra-specific variability tends to be lower than the divergence between species, but no barcoding gap is detected. The success rate of the statistical methods of species identification is excellent (up to 99% or 100% for statistical supervised classification methods as the k-Nearest Neighbour or Random Forest). The 16S gene is 2.5 less variable than the Cytb and CO1 genes. As a result its discriminatory power is smaller. To sum up, our results suggest that using DNA markers for identifying species in the Praomyini tribe is a largely valid approach, and that the CO1 and Cytb genes are better DNA markers than the 16S gene. Our results confirm the usefulness of statistical methods such as the Random Forest and the 1-NN methods to assign a sequence to a species, even when the number of species is relatively large. Based on our NJ trees and the distribution of all intraspecific and interspecific pairwise nucleotide distances, we highlight the presence of several potentially new species within the Praomyini tribe that should be subject to corroboration assessments.

## Introduction

The Praomyini tribe (Murinae subfamily) is one of the most diverse and abundant groups of Old World rodents. It is well defined on molecular grounds [1] and contains eight genera and more than 50 species. The systematics of this tribe has long been controversial due to the existence of many sibling species (i.e., species that are similar in appearance but are nonetheless reproductively isolated from each other). Fortunately, over the past decades, the development of molecular and/or morphometrical techniques has been extremely efficient in characterising Praomyini species and has progressively yielded a more comprehensive view of the systematics of this tribe [2]–[15]. However, in many papers, Praomyini species identification is still incomplete or erroneous [16]–[19]. This is important since several species are known to be involved in crop damage [20], [21], as well as in the epidemiology of several human or cattle diseases (e.g. plague [22], leptospirosis [23], Lassa hemorragic fever [24], [25], mycobacteria [26], [27]). Moreover Praomyni species are abundant in all habitats (forest, savannah, anthropised habitats) and generally represent more than half of the specimens captured [28]–[33]. Thus an easy, fast and accurate species identification tool is needed for non-systematicians (epidemiologists, agronomists, ecologists, etc) to correctly identify Praomyini species.

DNA barcoding could fulfil this need. DNA barcoding is a process that uses a short DNA sequence from a standard locus, i.e. the 5′ half of the cytochrome c oxydase I (CO1) mtDNA gene, as a species identification tool [34]. CO1-barcoding has been shown to provide sufficient resolution and robustness in some groups of organisms, such as arthropods, birds and fish [34]–[39]. Few studies on the CO1 gene have been conducted in mammals (see [40]–[44]), and DNA barcoding has never been tested in the Praomyini tribe. A good synthesis of the advances and limitations of DNA barcoding was recently published by Frézal and Leblois [45]. The cytochrome b (Cytb) has also been suggested as a marker to determine species boundaries in mammals within the framework of the genetic species concept [46]. A first study comparing the relative values of Cytb and CO1 for phylogenetic reconstruction and identification of mammalian species was recently published [47]. It showed that the Cytb gene more accurately reconstructs the mammalian phylogeny and gives better resolution for separating species. Comprehensive tests are still needed to confirm the most appropriate marker(s) to resolve species boundaries in rodents.

**Figure 1:**
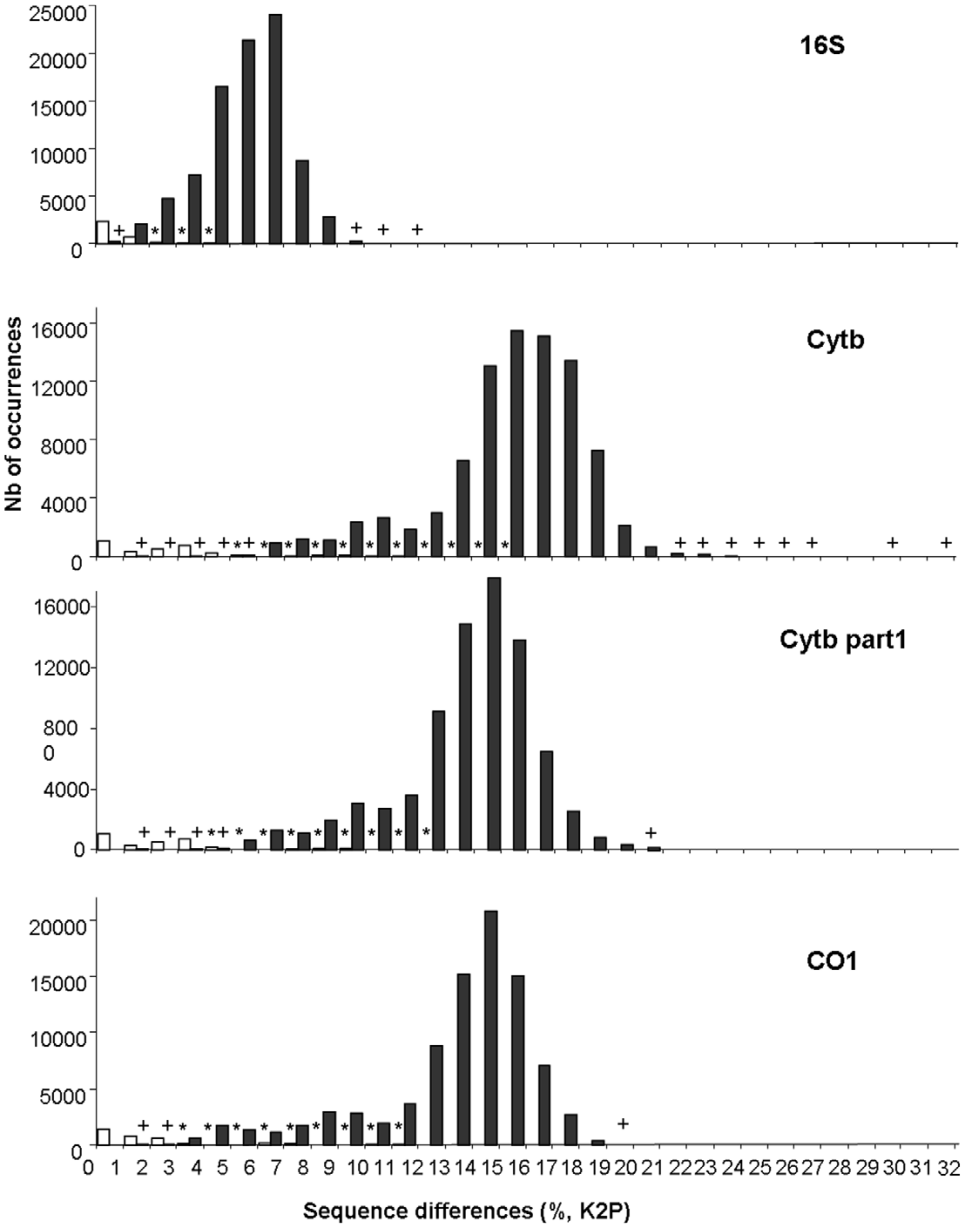
Distribution of intraspecific (white bars) and interspecific (black bars) divergences estimated from the K2P distance for the genes 16S, Cytb and CO1 and for the first part of the Cytb gene. In several cases a non-null number of occurrences was observed, but this is not apparent on the histograms because of the scale. The symbol “*” indicates a non-null number of occurrences within species, and “+”a non-null number of occurrences between species.

**Table 1:** Mean, minimum and maximum distances observed between individuals of the same (intraspecific) or distinct species (interspecific) for each gene.

	Intraspecific			Interspecific		
	mean	min	max	mean	min	Max
P distance						
16S	0.77	0.00	4.41	5.24	0.00	9.31
Cytb	2.92	0.00	14.42	13.56	1.36	25.12
Cytb part 1	2.49	0.00	10.00	12.27	1.04	18.98
CO1	2.89	0.00	14.29	12.00	1.00	16.90
K2P distance						
16S	0.78	0.00	4.56	5.46	0.00	10.01
Cytb	3.07	0.00	16.44	15.27	1.38	31.13
Cytb part 1	2.60	0.00	10.90	13.66	1.36	22.25
CO1	2.03	0.00	11.73	13.32	1.01	19.83

**Figure 2:**
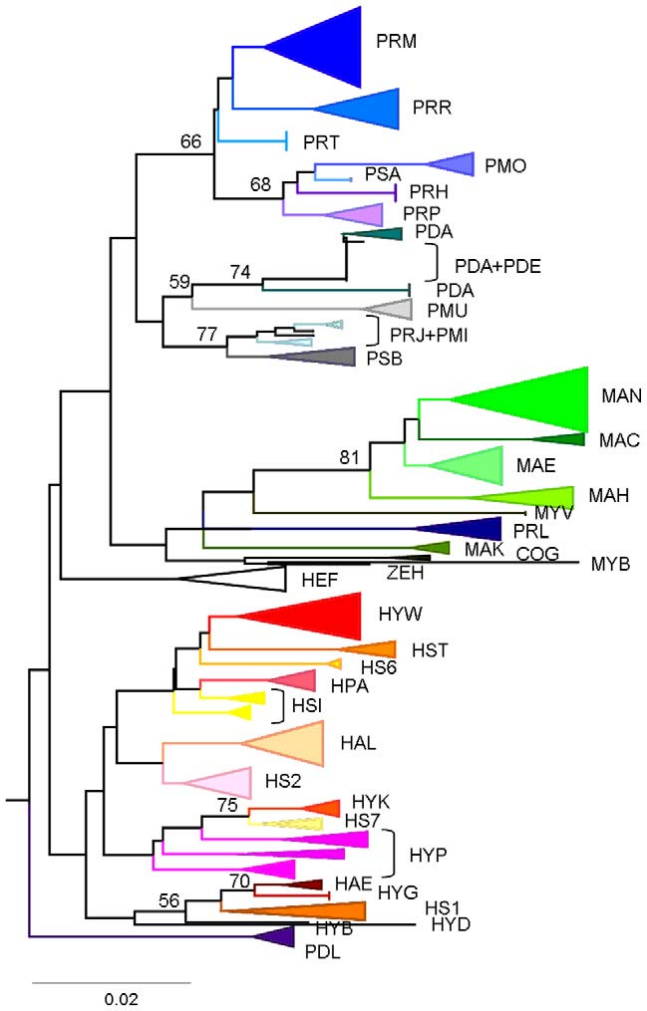
16S neighbour-joining tree of Praomyini (K2P distance), with bootstrap support (500 replicates). To improve clarity, bootstrap support of each species is not indicated on the tree but is reported in Table 1. For species codes, see Table S1.

**Figure 3:**
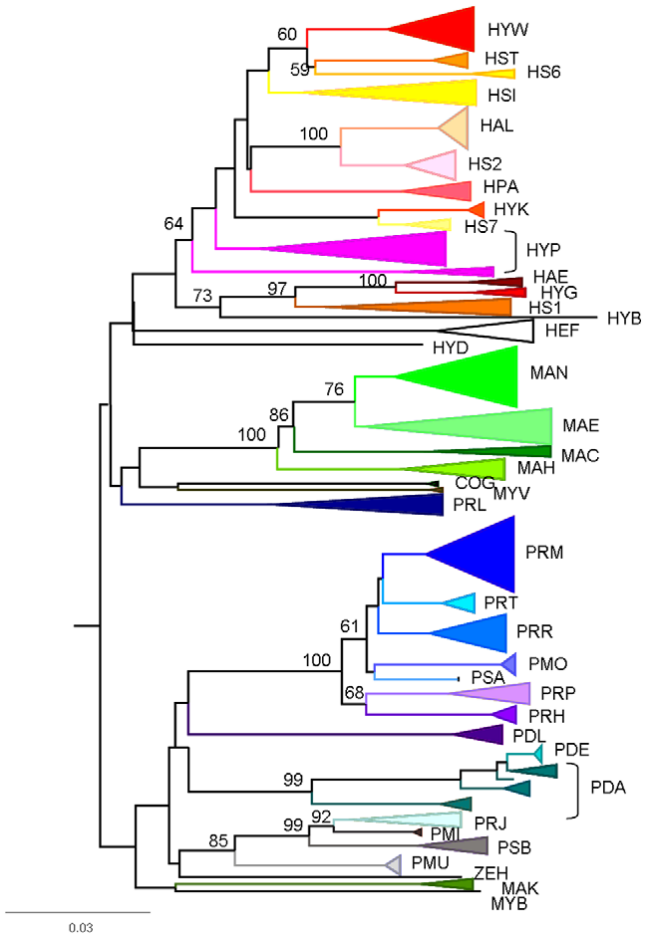
CO1 neighbour-joining tree of Praomyini (K2P distance), with bootstrap support (500 replicates). To improve clarity bootstrap support of each species is not indicated on the tree but is reported in Table 1. For species codes (see Table S1).

The most widely used mtDNA markers for resolving phylogenetic relationships and for inferring species boundaries in the Praomyini tribe are the 16S and Cytb genes [2], [3], [5], [7], [12], [14], [15], [48], [49]. Moreover, several species-level phylogeographic studies of this group based on the Cytb gene were recently published [50]–[53].

**Table 2:** Success rates (%) obtained by performing the two assignment methods (RF and *1*-NN) with the three genes (16S, Cytb, CO1) and the first part of the Cytb gene.

Gene	16S	Cytb	CO1	Cytb-part1
**RF**	97.87 [96.79–98.95]	99.53 [98.91–100]	100	99.52 [98.90–100]
**1-NN**_SM_	rand	99.29 [98.29–100]	100	100
	next	99.29 [98.57–100]	100	100
**1-NN**_K2P_	rand	99.29 [98.29–100]	100	100
	next	99.29 [98.57–100]	100	100

Confidence intervals (5%) are given in brackets.

In this study, we compare the usefulness of three genes (16S, Cytb, CO1) for identifying species in the Praomyini tribe. This makes it possible to test if the recommended DNA barcode region (CO1) is suitable for species identification in this tribe, which includes a large number of recently diverged species. According to Dasmahapatra and Mallet [54] many studies published on barcoding are biased because intraspecific variation has been underestimated (a small number of specimens sequenced per species from a restricted geographic area), whereas interspecific variation has been overestimated (closest relatives not included). This agrees with the results obtained in Austerlitz et al. [55] where the performances of all the methods are improved for an increased number of specimens per species (which allows the statistical algorithms to take intra and interspecific variations together with possible diagnostic mutations more effectively into account). To overcome these biases, we tried to include all of the species of the tribe, as well as specimens from the entire geographic range of each species. Several methods for analysing DNA sequences for the purpose of taxonomic assignment are commonly used (reviewed by [56] and [55]). First, it was shown that there was generally no best-performing method, i.e., a given method could perform better than another for a given evolutionary scenario, whereas the reverse could be true for another one [55]. Second, the parameter that had the most influence on the performances of the various methods was the data molecular diversity. To study the performance of the three genes for identifying species of the Praomyini tribe, we used a phylogenetic method (neighbour joining tree), and two supervised statistical classification methods: one is based on distance (k-nearest neighbour referred to as 1-NN), and the other one based on an impurity criterion (Random Forest referred to as RF). Finally, we investigated species boundaries. This is a long-standing problem and many methods based on DNA sequences have been proposed [57], [58]. Most of these methods rely on the presence of a “barcoding gap” (i.e., a genetic distance cut-off that could be used as an indicator of differentiation between species). Since there is no barcoding gap within the Praomyini tribe, we first used the approach of Meyer and Paulay [59] based on thresholds. We then proposed a simple approach based on the increase of intraspecific divergences.

**Figure 4:**
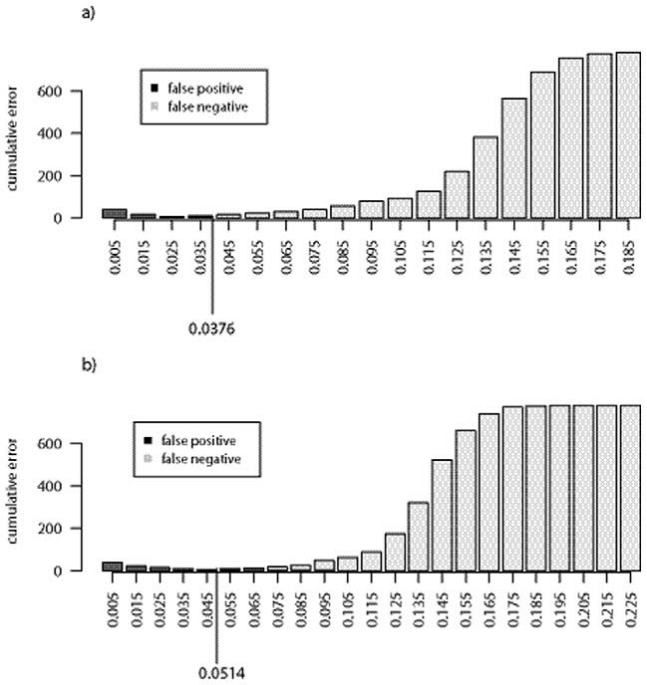
Distributions of the cumulative errors among the 40 species of Praomyini tribe, calculated from: (a) the CO1 gene; (b) the Cytb-part1 gene.

## Methods

Animals were live-trapped using Sherman traps (H.B. Sherman Traps, Inc.n FL U.S.A.) and handled under the guidelines of the American Society of Mammalogists (ASM; http://www.mammalsociety.org/articles/guidelines-american-society-mammalogists-use-wild-mammals-research-0; Animal Care and Use Committee, 2011). Trapped animals were euthanised by thoracic compression for smaller species and by the injection of a lethal dose of isofluorane, followed by cervical dislocation for bigger species. The protocol was approved by the French National Museum of Natural History (ATM Barcode 2010–2011, BQR Rayonnant 2004–2006) and by local authorities in concerned African countries (2003/PFHG/05/GUI: Ministry of Public Health, Republic of Guinea; 41/MINRESI/B00/C00/C40: Ministry of Scientific Research and Innovation, Cameroon; 158/07-C, 159/07-C: Ministry of Rural Development, Benin).

**Table 3:** Proportion (%) of the pairwise distances belonging to the 0.90^th^ and 0.95^th^ quantile of the distribution of the intraspecific pairwise distances.

		0.90th quantile				0.95th quantile			
Species	Nb of pairwise distances	CO1	Cytb	Cytb-part1	16S	CO1	Cytb	Cytb-part1	16S
HEF	66	0.00	0.00	0.00	0.06				
HS1	36	0.39	0.39	0.25	0.22	0.06	0.00	0.22	0.00
HSI	78	0.41	0.40	0.46	0.12	0.01	0.00	0.06	0.00
HYP	231	0.65	0.68	0.68	0.70	0.40	0.66	0.43	0.56
HYW	231	0.00	0.00	0.00	0.01				
MAE	153	0.08	0.00	0.00	0.12	0.02	0.00	0.00	0.00
MAH	55	0.00	0.00	0.00	0.09				
PDA	253	0.38	0.44	0.44	0.44	0.26	0.05	0.16	0.14
PRL	55	0.45	0.33	0.33	0.00	0.02	0.00	0.00	0.00

Only lines with non-zero elements are listed.

### Taxon sampling

Our study included seven of the eight genera of the Praomyini tribe (*Colomys, Zelotomys, Heimyscus, Hylomyscus, Mastomys, Myomyscus, Praomys, Stenocephalemys*). *Colomys* and *Heimyscus*, the two monotypic genera, were also represented. Five of the eight species of *Mastomys*, two of the four species of *Myomyscus* and one of the two species of *Zelotomys* were included. Musser and Carleton [56] recognised eight species in the genus *Hylomyscus*. However, two additional species have recently been described [6], [7], and a recent molecular study [5] suggested that the forms *kaimosae* and *simus*, considered as synonyms of *stella* and *alleni,* respectively, by Musser and Carleton, should be considered as distinct species. Moreover, the latter study underlined the existence of several undescribed species within this genus. In the present study, we used the nomenclature proposed by Nicolas et al. [5]. Our sampling includes all but one species of *Hylomyscus* (*H. carillus*), as well as four taxa representing candidate species based on unpublished molecular and morphometrical data (for a definition of candidate species see Padial and De la Riva [60]: populations for which there is some but incomplete evidence of species status and that have not received a formal name). Fourteen of the 17 *Praomys* species recognised by Musser and Carleton [61] were also included, as well as two new candidate species [62].

For each species, one to 37 specimens were sequenced (with an average of 11: see Table S1). Finally, 426 specimens were sequenced for the three genes. All specimens were identified by the specialist of the group using a combination of morphological, morphometrical and cytogenetical molecular data. Details on all specimens (sampling location, GPS coordinates, voucher number, BOLD number, etc.) are available within the ‘‘PRAOM” project in the Barcode of Life Data Systems (BOLD. www.barcodinglife.org).

### DNA extraction, amplification and sequencing

DNA was extracted from ethanol-preserved muscle, liver or heart by either the Cetyl Trimethyl Ammonium Bromide (CTAB) method [63] or by proteinase K digestion using the NucPrepTM chemistry isolation of a gDNA kit (Applied Biosystems, Courtaboeuf, France).

The Cytb gene was amplified using PCR primers L14723 (CCAATGACATGAAAAATCATCGTT), and H15915 (TCTCCATTTCTGGTTTACAAGAC) [64]. When DNA was degraded and amplification of the entire gene could not be achieved in one step, the internal primers L14749 (ACGAAACAGGCTCTAATAA) and H14896 (TAGTTGTCGGGGTCTCCTA) were used. The 16S gene was amplified using PCR primers 16SA (CGCCTGTTTAACAAAAACAT) [65] and Hm (AGATCACGTAGGACTTTAAT) [66]. The CO1 gene was amplified using the primers BatL5310 (CCTACTCRGCCATTTTACCTATG) and R6036R (ACTTCTGGGTGTCCAAAGAATCA) [41]. The PCR consisted of 35 cycles: 30 s at 94°C, 40 s at 48–55°C and 90 s at 72°C. The double-stranded PCR products were purified and sequenced at the Genoscope (Evry, France). The 16S gene generally presents insertions and its alignment is much more difficult than the other two genes. For this gene, sequences were aligned using Clustal [67], and the resulting matrix was then manually corrected. The final alignment comprised 510 nucleotides for the 16S gene, 1077 nucleotides for the Cytb gene and 697 nucleotides for the CO1 gene.

All sequences were entered into the BOLD database under the process-ID PRAO001-11 to PRAO437-11, and in the Genbank database (CO1: JQ667597-JQ668026; Cytb: JQ735467-JQ735889, JF343847, JF343852, JF343858, FJ617509, JF343860, JF343866, JF343850, JF343847, JF343847, JF343852, JF343858, FJ617509, JF343860, JF343866, JF343866; 16S: JQ843689-JQ844108, JF284175, JF284181, JF284182, FJ786196, FJ786177, JF284198, JF284177, JF284176, JF284184, JF284173).

### Data analysis

First, frequency histograms of the distribution of all conspecific pairwise distances and all heterospecific pairwise distances were constructed in order to look for the presence of a barcoding gap. The pairwise distances were computed with two methods: the p-distance or normalised Hamming distance (proportion of nucleotide sites at which two sequences being compared are different) and the K2P distance (Kimura, 1980).

Second, a tree-based approach of species delimitation was used. Since our aim was to provide a robust and rapid identification of taxa rather than an accurate determination of their phylogenetic relationships, we just needed “a fast and simple to use” tree building method (i.e. that could be used by a non-biologist or non-systematician). Hence, we used a phenetic (distance-based) tree-generating algorithm. Sequence divergences were calculated using the K2P distance model [68], and a neighbour joining (NJ) tree of K2P distances was created with PAUP 4b10 [69] to provide a graphic representation of the patterning of divergences among species [70]. Bootstrap analyses (500 replicates) were used to estimate the robustness of internal nodes. The tree-based criteria of reciprocal monophyly (a topological criterion that neither of two sister lineages be visually nested within the other) was used to define species boundaries (see [71] for a discussion on the limits of this criteria). Our phylogenetic trees were rooted with three distantly related outgroups, all belonging to the Murinae subfamily: *Malacomys longipes* (Malacomyini tribe), *Bandicota indica* (Rattini tribe) and *Rattus rattus* (Rattini tribe).

Third, statistical assignment methods 1-NN and Random Forest, were performed on each gene (or on parts of it) in a supervised classification framework detailed below (see, e.g., Clarke et al. [72] for a comprehensive text about all the statistical classification and clustering methods). The *k*-Nearest Neighbour classification assigns the status obtained from the majority vote among its *k* nearest neighbours to a query sequence [73]. Cover and Hart [74] have shown that, in some sense, half the classification information is contained in the nearest-neighbour (NN) and that among certain classes of distributions, the 1-NN rule is better than the k-NN rule. In addition, Austerlitz et al. [55] observed that for barcoding purposes, k = 1 provided better results than k = 2 or k = 3. Therefore, in this study, we used the *1*-NN rule based either on the p-distance or on the K2P distance. When two sequences with different statuses were located at the same distance from the query sequence, two procedures were used to select a status: the “rand” procedure that randomly assigns one of the two statuses, and the “next” procedure that assigns the status of the next nearest individual.

The Random Forest assignment method [75] is based on the “Classification And Regression Trees” algorithm (CART) [76] that consists in recursively constructing a binary tree according to the following rules. The root node contains all of the DNA sequences of the training set. At each step, a set (node) is partitioned into two subsets (sub-nodes) according to a splitting rule based on the allelic state of the reference sequences at a given site. The accuracy of each possible partition is computed according to its impurity 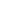, measured here by its Gini index: 
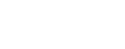
, where 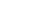 is the proportion of sequences belonging to species *j* at node *t* (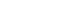). The impurity reduction obtained by splitting the sequences of node *t* into two sub-nodes *“s1”* and *“s2”* according to their allelic state at site *s* is expressed as 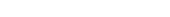. The site that provides the largest reduction is selected. The splitting process is stopped when the node is pure or when no additional node leads to a reduction of the impurity. Once the tree is built, each query sequence is assigned to a leaf of the tree according to its allelic state at the selected sites, and the query sequence is assigned to the majority species of the leaf. A known limitation of this CART algorithm is that it overweights the first splitting node. To overcome this fact and to improve the robustness of CART, the Random Forest algorithm constructs a family of trees from the training set by randomly choosing subsets of *m* polymorphic sites and running CART on these new training samples. The query sequence is then assigned to the species obtained by the majority of trees. As in Austerlitz et al. [48], *m* is chosen to be equal to the square root of the total number of the polymorphic sites.

To study the error rates (or performances) of these various methods, we preferred to use ten-fold cross-validation than the "leave-one-out" method. Indeed Cross-Validation is a standard tool for assessing model fit in a predictive accuracy sense. It is a compromise between the need to fit and the need to assess a model. A ten-fold Cross-Validation is performed as follows. The *n* observations data set is randomly split into ten partitions. The "learning set" (i.e., in this case, a set of reference sequences known to belong to the species of the tribe that have already been described) contains all but one of the partitions, referred to hereafter as the "test set" (i.e., in this case, a set of sequences with masked taxonomic status). Based on each learning set, a classification algorithm is first built and then used to assign a status (i.e., in this case, a species) to each individual of the test set. The result of the assignment is then checked against the unmasked taxonomic status and a misclassification rate is computed. The prediction error is assessed for each of the ten test sets and then averaged. The Leave-One-Out method is just a special case of Cross-Validation with only one observation successively removed from the data set. Indeed, Leave-One-Out yields an unbiased estimation of the true prediction error but can have high variance because the *n* training sets are so similar to one another (see, e.g., [72]). Hence, the results obtained with Cross-Validation are more reliable than L-L-O results since Cross-Validation automatically takes the various noise levels present in different data sets into account. Moreover, although statistical classification algorithms are designed to deal with within-group variability, they do not rely on its knowledge for their implementation. Therefore, groups containing few individuals can be included in the analysis.

The performance of each of the three genes was evaluated as the rate at which the query sequences were successfully assigned to their species. Confidence intervals for the probabilities of good assessment were simultaneously obtained using a ten-fold Cross-Validation procedure implemented for each gene and each method.

Before performing these statistical assignment methods, data sets were pre-treated: all values different from “a”, “c”, “g” and “t” were considered as missing data. All sites containing more than 10% of missing data were removed (e.g., site 5 of the Cytb gene). Finally, only the species including more than two individuals were kept.

Fourth, we investigated species boundaries. To do this, we first used the Meyer and Paulay [59] approach. In this framework, the assumption (H0) is “Two specimens belong to different species”, so that “False Negatives” are specimens coming from two different species that are classified within the same species (“Type I” error), and “False Positives” are specimens belonging to the same species that are classified in two different species (“Type II” error). “H0” is then accepted when the interspecific distance is greater than a threshold “t”. By varying the threshold “t” from 0 to the maximum of interspecific distances, we can draw the cumulative distribution functions of “False Positives” and of “False Negatives” as a function of the interspecific K2P distances. Meyer and Paulay [59] used the rate of these errors to suggest a minimisation of their sum in order to obtain an optimal threshold value. We first observed that differences between the numbers of intra- and interspecific divergences strongly influence the optimal threshold as defined by Meyer and Paulay. We therefore modified their method using the number of errors instead of the error rates. Finally, since there is no barcoding gap in the Praomyini tribe, methods based on the interspecific distances could not been used. We proposed to more precisely study the distribution of intraspecific pairwise distances in order to identify the species to which the individuals forming the tail (the *p* ^th^ quantile) belong: we chose the tail corresponding to the 0.90^th^ and .095^th^ quantiles for the three genes.

The Cytb gene is long (1077 bp retained for our study) and its complete sequence can only be obtained through two sequencing reactions. Thus, taking the cost of sequencing into account, it is interesting to investigate the performance of only the first part of this gene (obtained in one sequencing reaction). The first part was tested since it is used more often than the last part of the gene in phylogenetic and phylogeographic studies. Consequently, all of the analyses described above were performed both on the three genes (16S, CO1, Cytb) and on the first part of the Cytb (670 bp), referred to as Cytb-part1.

## Results

All of the genes investigated exhibited rather high mutation rates among the Praomyini tribe. For example, using the Watterson estimator compared to the improved estimator of Futschick and Gach [71b]) for theta, we obtained 29.2, 44.1, 83.9 and 48.9 (compared to 28.0, 42.4, 80.4 and 40.0) for the 16S, CO1, Cytb and Cytb-part1 genes respectively.

With the exception of a few interesting examples discussed below, sequence differences between species are far greater than sequence differences within species for all genes (Table 1). However, no barcoding gap could be detected (Fig. 1). Results obtained with the two distance methods (p-distance and K2P distance) were similar. Thus only the histograms obtained for the K2P distance are shown on Fig. 1.

Intra- and interspecific divergences are significantly higher for the Cytb and CO1 genes than for the 16S gene (p values <0.05). For all genes, the greatest intraspecific sequence divergences (K2P distances >2.04%, 7.50%, 8.80% and 9.87% for the 16S, CO1, Cytb, Cytb-part1 genes, respectively) are obtained for specimens of *P. daltoni* or of *H. parvus*. For all of the genes, a wide range of interspecific pairwise comparison values is obtained: the lower values (K2P distances <0.49%, 2.94%, 4.87% and 3.47% for the 16S, CO1, Cytb, Cytb-part1 genes, respectively) are always obtained between specimens of *P. daltoni* and *P. derooi*. Moreover, several identical sequences were obtained between specimens of these two last species for the 16S gene.

NJ trees built with the two distance methods (p-distance and K2P distance) were similar so only those obtained for the K2P distance are shown on Figs. 2–3 and S1 and S2. Trees obtained for the Cytb, Cytb-part1 and CO1 genes were similar. Thus only the tree obtained for the CO1 gene is shown on Fig. 3, whereas trees obtained for the Cytb and Cytb-part1 genes are presented as supplementary data (Figs. S1 and S2). With the exception of a few interesting examples discussed below, all species are monophyletic in the four gene trees. However, species bootstrap supports are higher for the Cytb, Cytb-part1 and CO1 dataset than for the 16S dataset (Table S1).

Deep divergences within *H. parvus* are observed for the three genes, and this species appears paraphyletic in the 16S (three groups), Cytb and CO1 (two groups) trees. *H. parvus* is monophyletic only in the Cytb-part1 dataset, but this clade is not supported (bootstrap value <50%).

Deep divergences occurred within *P. daltoni* which is paraphyletic with respect to *P. derooi* in the CO1, Cytb and Cytb-part1 trees. *P. daltoni* and *P. derooi* are polyphyletic in the 16S tree, but cluster together (bootstrap value: 74% with the K2P distance, and 71% with the p-distance).

*H. simus* is paraphyletic in the 16S gene tree, whereas it is monophyletic in the three other gene trees. However the distribution of all pairwise K2P distances shows a gap, regardless of the gene.

*P. jacksoni* and *P. minor* are also polyphyletic in the 16S gene tree, whereas they are monophyletic in the three other gene trees These two species cluster together in the 16S tree, but this clade is not supported (<50%). On the other hand, they cluster together with high bootstrap support in the three other trees.

Distance-based tree-generating algorithms are not suitable to infer phylogenetic relationships between species. Thus, we will not discuss results obtained above the species level in detail. However, it is interesting to note that five clades are recovered in all of the analyses: one clade included four of the five *Mastomys* species (*M. coucha*, *M. erythroleucus*, *M. huberti* and *M. natalensis;* the fifth species, *M. kollmanspergeri,* has an unstable position in the tree); one clade includes *P. jacksoni*, *P. minor* and *Praomys* spB; one clade includes *P. misonnei*, *P. rostratus*, *P. tullbergi*, *P. morio*, *Praomys spA*, *P. hartwigi* and *P. petteri*; one clade includes *H. aeta* and *H. grandis*; and one clade includes *H. kaimosae* and *Hylomsycus* sp7. More nodes are supported in the Cytb, Cytb-part1 and CO1 trees than in the 16S tree and they are largely congruent between genes.

The results of the two assignment methods (Random Forest and 1-NN) performed on the three genes (16S, Cytb and CO1) and on Cytb-part1 are presented in Table 2. The CO1 gene shows 100% of well classified individuals regardless of the assignment method. The Cytb gene also leads to 100% of correct assignment when using the *1*-NN method. However, when Random Forest is used the performance slightly declines to an average of 99.53% with a 95% confidence interval going from 98.91 to 100. The first part of the Cytb gene performs as well as the entire gene, regardless of the assignment method. When the 16S gene is used, the well-classified rates decrease to an average of 99.29 and 97.87 with the *1*-NN and RF methods, respectively. Moreover, the 95% confidence interval calculated with the RF method does not contain the 100% value. All misclassified specimens (seven specimens) belong to *P. derooi* and they all were assigned to *P. daltoni*. The opposite occurs in the *1*-NN method where all misclassified specimens (three specimens) belonging to *P. daltoni* were assigned to *P. derooi*.

Given the previous results, we used the CO1 and Cytb-part1 genes to explore species boundaries using the Meyer and Paulay [59] approach. The distributions of false-positives and false negatives calculated for each gene are represented on Fig. 4. With the CO1 gene, the sum of errors is minimised for the threshold value of 0.0376. This value indicates three 3 false positives “HYP” (mean K2P distance  = 0.0639), “PDA” (0.0421) and “HSI” (0.0405) and one false negative “HS7-HYK” (0.0346). With the Cytb-part1 gene, the sum of errors is minimised for the threshold value of 0.0514. This value indicates one false positive “HYP” (0.0901), and two false negatives “PDA-PDE” (0.0466) and “PMI-PRJ” (0.0492).

Both genes lead to the same false positive *H. parvus*. Using the HAC technique we explored the proximities of specimens belonging to this species. Resulting dendrograms are given in Fig. S3. Cutting the “CO1- dendrogram” of *H. parvus* at the threshold level (0.0376) leads to three groups with the maxima of intra-group variabilities lower than 0.0280 and inter-group divergences higher than 0.0697. Cutting the “Cytb-part1 dendrogram” at the threshold level (0.0514) leads to similar results except for one specimen (HYP_G10022) that merges at 0.0677 with one of the three groups. The false positives “PDA” and “HSI”, revealed by the CO1 gene were also investigated with HAC. For both species, cutting the dendrogram at the threshold level leads to two groups (Fig. S4).

The interspecific divergence “HS7-HYK” (0.0346) was revealed to be a false negative by the CO1 gene. Indeed, this value is low but the highest intraspecific pairwise difference (0.0190) remains considerably lower than the smallest inter-specific pairwise difference (0.0294).

Two false negatives, “PDA-PDE” and “PMI-PRJ”, were revealed by the Cytb-part1 gene.

HAC performed with *P. daltoni* and *P. derooi* species together shows that *P. derooi* is very close to one of the *P. daltoni* groups previously mentioned (Fig. S5). However the maximum of the “PDE” intra-specific pairwise differences is very low (0.0025), meaning that *P. derooi* is a very compact group.

HAC was also performed with *P. minor* and *P. jacksoni* species together. The dendogram obtained (Fig. S6) shows that the two species merge at a height slightly lower than the threshold.

Taking the lack of a barcoding gap into account, we investigated species boundaries by closely studying the tail of the intraspecific pairwise distance distribution. Results obtained for the 0.90^th^ and 0.95^th^ quantiles with the three genes and Cytb-part1 are presented on Table 3. The number of pairwise distances located in the quantiles is expressed as a function of the total number of pairwise distances within each species. At quantile 0.9, more than two-thirds of the values of “HYP” are located in the tail for all genes. With CO1 and Cytb genes, more than one-third of the values of “PDA”, “HSI”, “HS1” and “PRL” are located in the tail. At quantile 0.95, almost half of the values are still in the tail for “HYP”, whereas it decreases for the other species. Since we has already focused on “HYP”, “PDA” and “HSI”, we drew HAC dendrograms for the two other species (Fig. S7). With both genes, the two species showed two groups that merged above their respective CO1 and Cytb-part1 thresholds (as defined by the Meyer and Paulay approach).

## Discussion

### DNA-based species identification is possible for the Praomyini tribe

To be applicable to a particular group of species, DNA-based species identification requires no haplotype sharing between non-conspecific specimens. Haplotype sharing between species due to incomplete lineage sorting only occurred once in our 16S dataset: several specimens of *P. daltoni* and *P. derooi* have identical sequences. However this problem did not occur with the other two genes (Cytb and CO1) due to their higher evolutionary rate (more than 2.5 times higher).

Given that (1) nearly all the species included in our study are monophyletic in the NJ trees, (2) the degree of intra-specific variability tends to be lower than the divergence between species, (3) the success rate of the statistical methods of species identification is excellent (up to 99% or 100% for statistical supervised classification methods as KNN or RF), we can conclude that the presence of a barcoding gap is not necessary and that DNA-based species identification in the Praomyini tribe is a largely valid approach.

Our results confirm that this method is not only a powerful tool to assign a specimen to a species, but also to make it possible to look for new cryptic species. Nevertheless, a clear concept of what species are is required before trying to recognize and /or describe species. Despite the long history of diasgreement over species concepts, most species concepts hold that species are lineages of reproductive populations (evolutionary species concept; see de Queiroz [77] and Padial and de la Riva for a review [60]). Previous authors have generally disagreed about the best criteria for recognising these lineages. According to the evolutionary species concept, any organismal traits that evolved as a result of the independent trajectory of the reproductive population to which the organism belong can be used to propose a species hypothesis. Thus, DNA sequences can be relevant for discovering species because we can infer gene genealogies indicative of the historical processes that have divided lineages [78]. However, it should be mentioned that crucial pitfalls also exist [45]. All our results (NJ and HAC trees, frequency histograms, threshold methods) congruently indicate the presence of a cryptic diversity within *H. parvus* (probably three species instead of one) and *P. daltoni* (two species). A possible cryptic diversity within *P. daltoni* was also previously suggested by Bryja et al. based on molecular grounds [49]. According to our thresholds and HAC analyses *Hylomyscus sp1, H. simus* and *P. lukolelae* might also each represent a complex of 2 cryptic species. However, the low number of specimens available does not allow us to draw a conclusion. Moreover, for *Hylomyscus sp1,* two sub-clades in th NJ tree that cluster with low to high bootstrap support, depending on the gene considered, have been identified. To sum up, our results suggest the existence of several possible new species. These are only preliminary species hypotheses that should be tested using other types of traits (morphology, morphometry, cytogenetic data, etc) before we are really able to describe them.

### Comparative performance of the three mt DNA markers for identifying Praomyini species

The 5′ half of the CO1 mtDNA gene was proposed as the standard barcode. However, the mitochondrial genome is not suitable for plant DNA barcoding [79], [80]. For mammals, it was recently proposed that the Cytb gene would provide a better resolution for separating species than the CO1 gene [47]. According to Austerlitz et al. (2009), the most important parameters for species barcoding are those that determine the molecular diversity. This might vary considerably among genes and groups of organisms.

A suitable genetic marker for species identification within the Praomini tribe needs to meet a number of criteria. First, it must be flanked by conserved regions that can be used to develop universal primers. Second, sequence alignment should be easy and unambiguous (which is essential for the statistical methods to perform well). Third, the lack of heterozygosity that enables direct polymerase chain reaction (PCR) sequencing without cloning is an important criterion. Fourth, it should simultaneously contain enough variability to be informative for identification and be short enough to be sequenced in a single reaction. We will now review these four conditions for the three markers (16S, CO1, Cytb) tested in our study.

The primers used for the three genes were effective for all Praomyini, and are also routinely used to sequence other groups of rodents [41], [64], [81]–[84]. However, on several occasions, we amplified a Cytb nuclear pseudogene, which could be easily identified due to the presence of indels or diagnostic mutations (stop codons). The 16S gene presented some alignment difficulties due to the presence of insertions and deletions. The three genes tested in this study fulfil the third need (lack of heterozygosity), since the mitochondrial genome is haploid (maternally inherited).

It is largely accepted that the accuracy of species delineation depends on the extent of, and separation between, intraspecific variation and interspecific divergence in the selected marker. The more overlap there is between genetic variation within species and divergence separating sister species, the less effective barcoding-like method becomes. Several authors have argued that a “barcoding gap” exists between intra- and interspecific variation [36], [85]. However, others have shown that the gap was due to an underestimation of intraspecific variation (low number of specimens sequenced per species) and an overestimation of interspecific divergence (closely related taxa not included) [59], [86]. Our results clearly show that even when sampling is sufficiently comprehensive to robustly evaluate intra- and interspecific variations (comprehensive geographical and taxonomic sampling), there is an overlap between them. Indeed, an overlap exists for the three genes tested in our study. A small part of this overlap may be due to taxonomic problems (cryptic diversity). However, this overlap persists when we take the presence of cryptic species into account (Fig. S8). Hence, even in the absence of a “barcoding gap” for the three genetic markers tested in this study, our results show that they contain enough variability to be informative for species identification. According to our data, the 16S gene is 2.5 times less variable than the Cytb and CO1 genes. As a result its discriminatory power is smaller: (1) shared haplotypes between distinct species were observed; (2) a non-negligible number of interspecific sequence divergences were lower than 1%; (3) the number of non-monophyletic species was greater and the bootstrap support of species was smaller than for the two other genes; and (4) the percentage of correct classification in statistical methods was lower.

Owing to the length of the sequences analysed here (510, 1077 and 697 nucleotides for the 16S, Cytb and CO1 genes, respectively), only the 16S and CO1 genes could be sequenced in a single reaction. We therefore also performed all the analyses considering only the first half (670 bp) of the Cytb gene, and obtained similar results.

To sum up, our results suggest that the CO1 gene and the first half of the Cytb gene are better markers for identifying Praomyini species than the 16S gene. Thus our study confirms that DNA barcoding has great appeal as a universally applicable tool for identification of species, possibly even in automated handling devices [87]. We do not agree with the study of Tobe et al. showing that the Cytb gene would be better than the CO1 gene for separating species [47]. Their study had several drawbacks: (1) as acknowledged by the authors themselves, “it was assumed that species designations were accurate, although it is possible that errors may have occurred”; and (2) assessment of intraspecific variation was only performed on three species (human, domestic cattle and domestic dogs). Moreover, the study of Clare et al., based on the sequencing of 9076 individuals from 163 species of neotropical bats, showed that the CO1 gene is a powerful marker for species identification [44]. A taxon-by-taxon approach that includes a large number of specimens of closely related species identified by the specialist of the group is clearly indispensable to draw a conclusion about the relative performance of several genetic markers for species identification. A number of authors have suggested using several complementary genes for species identification [80], [88]. The degree of variability and the phylogenetic signal of the Cytb and CO1 genes are similar. Thus, according to our results a 670 bp-long (and even a 350 bp-long) long fragment of the Cytb or CO1 gene is sufficient to identify Praomyini species. However, because these two genes are maternally inherited (mitochondrial genes), hybrids cannot be detected through the sequencing of these genes. Mitochondrial introgression following hybridisation has been widely inferred, and can lead to inaccurate species identification when mtDNA barcodes are used [89]. According to bibliographical data, the only known example of mitochondrial introgression in the Praomyini tribe is found between the species *P. derooi* and *P. daltoni* and could be explained by past hybridisation followed be back-crosses with paternal lineages [49]. As already acknowledged by several authors [44], [45], [55], it would be interesting to sequence several nuclear genes to further investigate the extent of hybridisation in the Praomyini tribe. To do this it is still necessary to search for nuclear introns with a sufficient amount of variability to identify closely related species.

### DNA-based methods of species identification

Our results show that even in the absence of a barcoding gap, barcoding-like methods can perform very well. The choice of a simple distance or a K2P distance did not change the results. Statistical methods such as Random Forest and the 1-NN method are very rapid and efficient to identify Praomyini species. Our results confirm that the 1-NN method is one of the most effective [55]. This method merely states that the query belongs to the same species as the closest sequence, using a specific genetic distance. According to Austerlitz et al. [55] the best performance of the 1-NN method could be due to the fact that classification methods such as RF might be misled either by mutations shared between species, a common phenomenon observed in young species, or just because different young species do not possess enough inter-molecular variability. However, this drawback of RF could be easily overcome by trying to include more specimens of these species. Since many Praomyini species arose recently (speciation events within *Praomys* species complexes occurred during the Pleistocene) [2], [14], [62], some mutations are specific but are not yet diagnostic, which could explain the good performance of the 1-NN method. The statistical methods used in this paper are efficient for identifying known Praomyini species, but they are not suitable for detecting new undescribed species. NJ phylogenetic trees are useful for this purpose. The species *H. parvus* and *P. daltoni* are both polyphyletic in our NJ trees suggesting the presence of several cryptic species within each species. The distribution of all intraspecific and interspecific pairwise nucleotide distances can also be used to pinpoint new species: the greatest intraspecific sequence differences were obtained between specimens of *P. daltoni* and of *H. parvus*. Several authors have proposed using a threshold for species diagnosis [34], [36], but this idea has been refuted by others [44], [59]. Therefore, before setting thresholds, it would be judicious to focus on possible positive or negative errors from various diagnostic tools. When there is no clear barcoding gap, a simple method consists in identifying the groups of specimens that are heterogeneous with respect to their DNA sequences measured in one or several genes. This is performed by looking for the specimens that belong to the alpha-quantile (e.g., alpha  = 0.95 or 0.90) of the intraspecific pairwise distribution. Varying the quantile level could be used as a cursor to give taxonomists different points of view of the groups of specimens under study. As already reported by Padial and De la Riva [60], minimum levels of divergence for certain traits (including genetic divergence) cannot be demanded for species recognition under the evolutionary species concept. However, some simple tools can provide preliminary species hypotheses that should be subject to corroboration assessments.

## Supporting Information

Figure S1 **Cytb neighbour-joining tree of Praomyini (K2P distance), with bootstrap support (500 replicates).** To improve clarity bootstrap support of each species is not indicated on the tree but is reported in Table 1. For species codes, see Table S1.
(EPS)

Figure S2 **Cytb-part1 neighbour-joining tree of Praomyini (K2P distance), with bootstrap support (500 replicates).** To improve clarity bootstrap support of each species is not indicated on the tree but is reported in Table 1. For species codes, see Table S1.
(EPS)

Figure S3 HAC dendrograms of *H. parvus* built from (a) the CO1 gene and, b) the Cytb-part1 gene.
(EPS)

Figure S4 HAC dendrograms built from the CO1 gene of (a) *P. daltoni* and (b) *H. simus*.
(EPS)

Figure S5 HAC dendrograms built from the Cytb-part1 gene for *P. daltoni* plus *P. derooi.*
(EPS)

Figure S6 **HAC dendrograms of** ***P. minor*** **plus** ***P. jacksoni*** *:* (a) built from the CO1 gene; (b) built from the Cytb-part1 gene.
(EPS)

Figure S7 HAC dendrograms built from the CO1 (a, c) and Cytb-part1 (b, d) genes for (a-b) *H. sp1* and (c–d) *P. lukolelae*.
(EPS)

Figure S8 **Distribution of intraspecific (white bars) and interspecific (black bars) divergences estimated from the K2P distance** for the CO1 gene, taking cryptic species into account. In several cases, a non-null number of occurrences was observed (symbol x for intra-specific comparisons, and symbol + for inter-specific comparisons), but this is not apparent on the histograms because of the scale.
(EPS)

Table S1 **Number of specimens of the Praomyini tribe per species, with geographical coverage and species codes used in** **Figs.** **2** **–** **3** **.** C  =  complete geographical coverage; M  =  most of the geographical range of the species covered; P  =  partial geographical coverage, unknown  =  the distribution range of this species is still unknown. Bootstrap values (500 replicates) obtained for all species and analyses are indicated. P  =  polyphyletic; Pa  =  paraphyletic.
(XLS)
